# An Underestimated Factor: The Extent of Cross-Reactions Modifying APIs in Surface-Modified Liposomal Preparations Caused by Comprised Activated Lipids

**DOI:** 10.3390/molecules25194436

**Published:** 2020-09-27

**Authors:** Max Sauter, Jürgen Burhenne, Walter E. Haefeli, Philipp Uhl

**Affiliations:** Department of Clinical Pharmacology and Pharmacoepidemiology, Heidelberg University Hospital, Im Neuenheimer Feld 410, 69120 Heidelberg, Germany; juergen.burhenne@med.uni-heidelberg.de (J.B.); Walter-Emil.Haefeli@med.uni-heidelberg.de (W.E.H.); Philipp1.Uhl@med.uni-heidelberg.de (P.U.)

**Keywords:** liposomes, surface modification, API-modification, head group-functionalized phospholipids, macromolecular drugs, cross-reaction

## Abstract

Despite the nowadays available plentitude of strategies to selectively introduce functional surface modification of liposomes, in preclinical research this process is still primarily performed after liposomal preparation utilizing comprised activated phospholipids with functionalized head groups. However, because these activated lipids are present during the liposomal preparation process, they can cross-react with incorporated drugs, especially the particularly often utilized active esters and maleimide groups. Macromolecular drugs, being composed of amino acids, are particularly prone to such cross-reactions due to their often multiple reactive functionalities such as amino and disulfide groups. To demonstrate this impact on the formulation in liposomal surface modification, we assessed the extent of cross-reaction during the liposomal preparation of two activated phospholipids with typically used head group functionalized phospholipids, with the two peptide drugs vancomycin and insulin comprising disulfide and amino functionalities. Both drugs revealed a considerable fraction of covalent modification (estimated 2 to 12%) generated during the liposome preparation process with comprised activated lipids. Modification of the active pharmaceutical ingredients (APIs) was determined by high-resolution mass spectrometric analysis. These findings clearly demonstrate the non-negligibility of potential cross reactions using the post preparation liposomal surface modification strategy in preclinical research.

## 1. Introduction

Functionally surface-modified liposomes, with conventional PEGylated liposomes left aside, have become a popular tool for a variety of applications in drug delivery such as cancer targeting as well as oral and intracellular delivery [1,2,3,4,5]. Notable examples include liposomes modified with RGD-peptides for targeting the integrin αVβ3 expressed during neoplastic angiogenesis [6], antibody-modified liposomes for epidermal growth factor receptor targeting [7], liposomes modified with cell-penetrating peptides for intracellular drug delivery of intrinsically non-cell-permeable drugs [8], combinations thereof, and a variety of further applications [9]. The plentitude of applications can be traced back to the easy-to-handle process of liposomal modification.

Due to the authorities’ requirements on the unity of liposomal formulations, preparation under regulatory compliance must (and will) avoid side reactions or variance on the amount of bilayer components including the surface modification. In contrast, in preclinical research and formulation development, the ease of preparation is often of primary concern. Therefore, expeditious manufacturing methods are enjoying continued popularity. Because of this discrepancy, the transfer of preclinical results into regulatory settings may be hampered.

**Figure 1:**
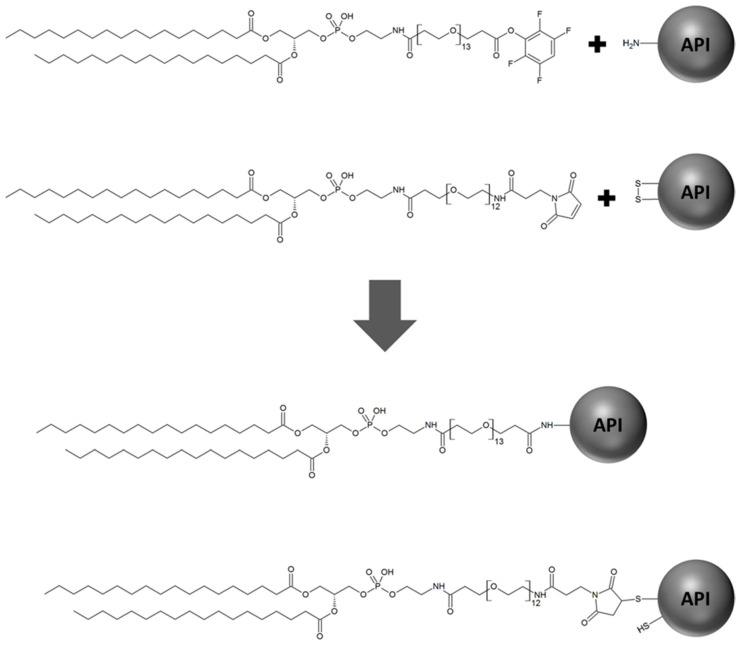
Scheme of the possible cross-reaction of activated ester and maleimide groups of phospholipids with amino and disulfide groups of active pharmaceutical ingredients (APIs).

In principle, surface modification of liposomes can be achieved either after liposomal preparation or by introducing the modification during the preparation process [9,10]. To introduce a surface modification during (or prior to) the preparation, the “conjugation agent” is mostly covalently linked to head group-modified phospholipids. This modification strategy exhibits the benefit, that it enables the purification of the conjugates prior to insertion avoiding unspecific and uncontrolled reactions. Examples for this surface modification strategy are, e.g., presented in the study of Kale et al. [11], Torchilin et al. [12] and de la Fuente-Herreruela et al. [13]. Nevertheless, due to the fact that this modification strategy requires high efforts (e.g., time-consuming, high personnel expenditure, specialized equipment for purification steps, requirement of sensitive analytical methods), the majority of liposome surface modifications in preclinical research are introduced via the incorporation of head group-activated phospholipids in the liposomal composition, which can be coupled to the modifying substances after the liposomal preparation process. While this modification strategy still represents the primary method utilized to prepare surface-modified liposomes [14,15,16,17], an important, often neglected impact is the risk of cross-reactions caused by the head group-activated lipids modifying the encapsulated active pharmaceutical ingredient (API) as depicted in Figure 1.

One further, and in our view improved, surface modification strategy represents the so-called post insertion technique reported previously, and, e.g., applied in a study of Biswas et al. [18]. In this study, the liposomal surface was modified by an R8 conjugate via spontaneous micelle-transfer technique. This technique did not alter liposomal main characteristics (size, PDI) while the successful integration of the peptide-PEG-phospholipid conjugate could be demonstrated by high increase in zeta potential. Further studies based on this strategy, to introduce conjugated phospholipids into pre-formed liposomes can be found in literature [19,20]. Moreover, click chemistry strategies that are targeting specific reactive groups have been established to prevent side reactions [21] due to the circumvention of reactivity towards active functional groups usually present in peptide therapeutics. However, click-chemistry may still exhibit reactivity to unnatural amino acids or modified side chains of some macromolecular therapeutics and can contribute to compositional variance of thus prepared liposomal formulations due to possibly incomplete reaction.

In contrast, surface modification of liposomes by PEGylated phospholipids without further functionalization, one of the most successful strategies to improve the delivery of therapeutic molecules [22], is not prone to potential cross-reactions. This may be one of the reasons why such liposomal formulations have been successfully implemented in clinical practice, e.g., Doxil^®^. The absence of further chemical reactivity makes PEGylation an easily applicable modification procedure. More importantly, due to the commercial availability of PEGylated phospholipids, additional coupling steps are redundant. However, with respect to clinical application, PEGylation also shows minor drawbacks, e.g., the cause of hypersensitivity reactions [23]. These observations force again the search for alternative strategies, e.g., the use of polysarcosine. In a previous study, polysarcosine was described as an alternative to PEGylation by incorporation into the bilayer of preformed liposomes via a post insertion technique [24].

With respect to recent efforts of incorporating peptides and proteins into functionally surface-modified liposomes, it has to be kept in mind that these substances mostly contain reactive amino acid residues such as disulfide (or thiol), hydroxyl, carboxyl, and amino functions. Up to date, there has been no universal assessment of the extent of API-modification occurring during liposomal preparation with distinct activated lipids. Therefore, the extent of this unwanted modification of the API is still unknown. Moreover, it is highly dependent of the APIs’ characteristics.

Recently, surface-modification has been introduced by prior coupling of the respective agent to head group-functionalized phospholipids before liposomal preparation [25]. However, possibly remaining activated lipids still pose a risk with respect to potential side reactions and have to be accurately separated prior to liposomal preparation and the encapsulation of APIs. One further approach rarely applied is to quench the reactivity of the head group-modified phospholipids by saturation with auxiliary materials, for example, cysteine for maleimide-reactive head groups [26]. Nevertheless, this may yield an insufficiently defined liposomal composition.

To demonstrate the impact of API modification occurring in the preclinical preparation of surface-modified liposomal delivery systems utilizing the post preparation modification strategy, we estimated the extent of the cross-reaction during the liposomal preparation process between two peptide drugs, namely the glycopeptide antibiotic vancomycin and the antidiabetic agent insulin, with two head group-modified phospholipids reactive towards thiol and amino functions. These two substances were selected due to their frequent use as model peptide drugs to assess novel liposomal systems, especially for oral drug delivery [27,28,29,30]. Modification of these peptide drugs was determined by high-resolution mass spectrometric analysis.

## 2. Results

The incorporation of activated lipid did not noticeably affect liposomal characteristics. Both activated lipids were incorporated in an amount of 1 mol-% without changing the size, polydispersity index (PDI), and lamellarity compared to control liposomes consisting solely of lecithin and cholesterol. Furthermore, no change in characteristics occurred when activated lipids were incorporated in liposomes containing the model compounds vancomycin or insulin (Table 1). 

The lipid-to-drug ratio of the vancomycin containing liposomes was 7.2 while the ratio was 360 for insulin containing liposomes. Due to the fraction of 1 mol-% of activated lipid in the liposomal bilayer components, these values correspond to molar activated lipid-to-drug ratios (Tfp-dPEG_13_-DSPE) of 0.036 and 7.1 for vancomycin and insulin containing liposomes, respectively (Table 2).

These different lipid-to-drug ratios were used as we aimed to adjust on different characteristics such as solubility of the macromolecular therapeutics. Vancomycin is highly soluble in neutral aqueous media while insulin is only slightly soluble at neutral pH and was predissolved in acidic conditions (in our case diluted hydrochloric acid 0.1 M). Therefore, we used a concentration of 50 mg/mL for preparing the vancomycin containing liposomes, which corresponds to a final concentration in the formulation of 10 mg/mL (6.9 mM), and for the insulin containing liposomes a concentration of 1 mg/mL was utilized corresponding to a final concentration of 0.2 mg/mL (0.035 mM). After the liposomal preparation process by dual asymmetric centrifugation, which was performed at room temperature and took 45 min in total, the liposomes were dissolved and the mixture directly infused into a Waters Xevo G2-XS QTof mass spectrometer for the detection of APIs and possible derivatives thereof.

While APIs encapsulated into control liposomes showed exclusively their original identities, APIs encapsulated into liposomes containing activated lipids contained substantial amounts of lipid-API-conjugates.

**Figure 2:**
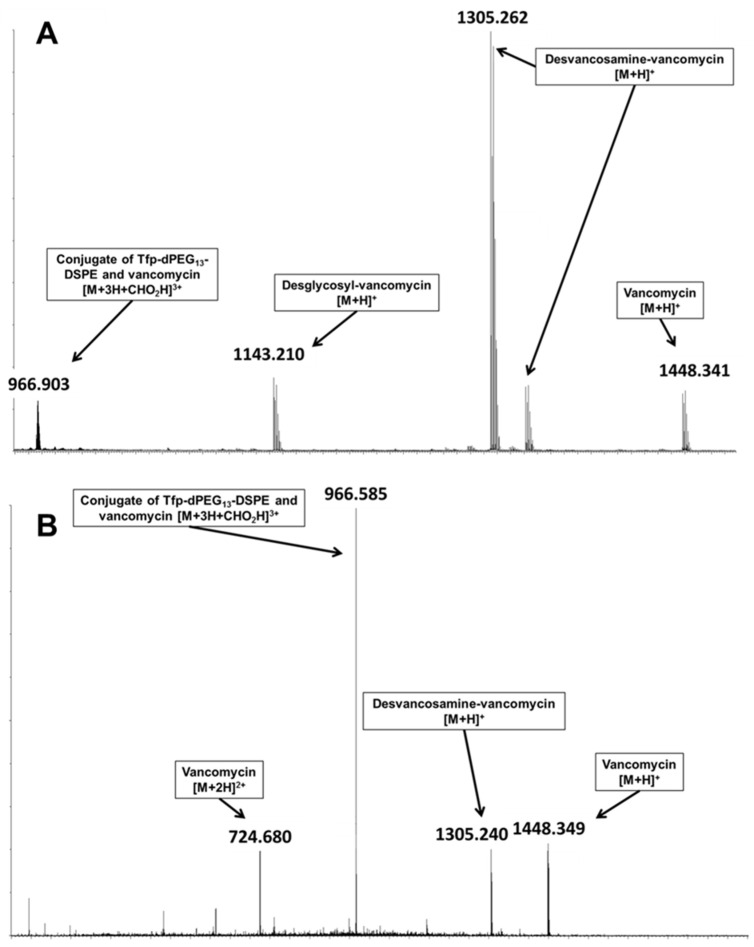
High-resolution mass spectrometric characterization of liposomes comprising Tfp-dPEG_13_-DSPE and encapsulated vancomycin. (**A**) Mass spectrum of vancomycin-containing liposomes comprising Tfp-dPEG_13_-DSPE. Visible is the formic acid mono-adduct of the conjugate of Tfp-dPEG_13_-DSPE and vancomycin ([M+3H+CHO_2_H]^3+^), while the remaining signals correspond to unmodified vancomycin with three being in-source fragments (desglycosyl-vancomycin, desvancosamine-vancomycin, and its corresponding sodium adduct). (**B**) Positive product spectrum of the [M+3H+CHO_2_H]^3+^ signal of the conjugate of Tfp-dPEG_13_-DSPE and vancomycin after collision-induced dissociation (CID) at 30 V. Besides the parent signal, the primary CID fragments are ions corresponding to vancomycin confirming the conjugates identity.

For vancomycin, encapsulated in Tfp-dPEG_13_-DSPE-containing liposomes, the formic acid adduct of the conjugate of Tfp-dPEG_13_-DSPE and vancomycin was detected (Figure 2A) with a monoisotopic *m/z* of 2894.672 (calculated 2894.351). Additionally, the collision-induced dissociation of the signal primary yielded ions of vancomycin (Figure 2B) as product ions, thus confirming the conjugate‘s identity.

Although ionization characteristics may differ between the conjugate and vancomycin, the detected signal indicates substantial derivatization of the liposomal encapsulated vancomycin, with an estimated modified fraction of 2%. The modified fraction was determined by comparison of the intensities of the respective most intense signals ([M+2H]^2+^ for vancomycin and [M+3H]^3+^ for the conjugate).

**Figure 3:**
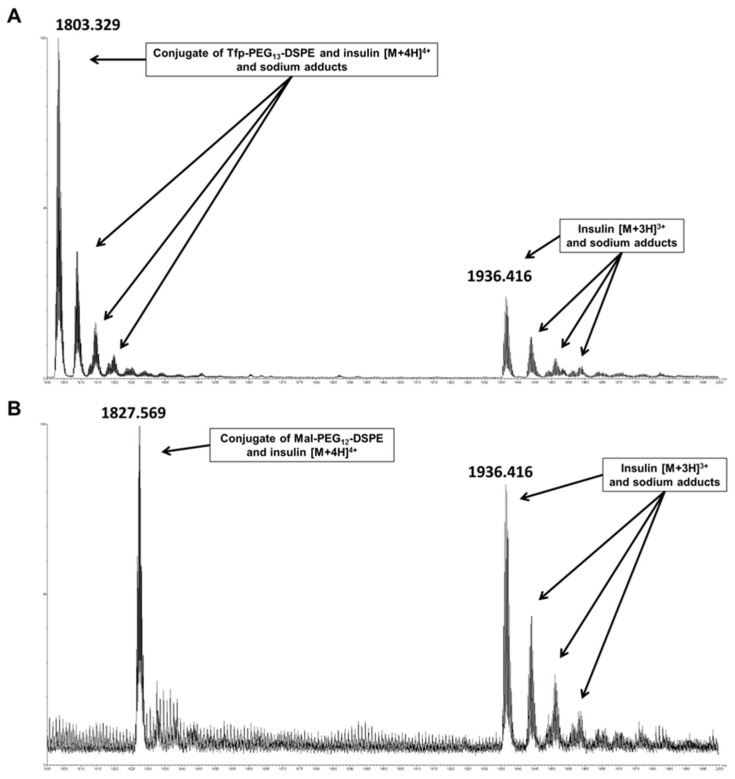
High-resolution mass spectra of liposomal formulations of insulin comprising lipids with maleimide and activated ester functions. (**A**) Liposomal formulation of insulin containing Tfp-PEG_13_-DSPE. Visible are the [M + 3H]^3+^ ion of insulin with its sodium adducts and the [M + 4H]^4+^ signal of the conjugate of insulin and Tfp-PEG_13_-DSPE with the corresponding sodium adducts. (**B**) Liposomal formulation containing Mal-PEG_12_-DSPE. Visible are the [M + 3H]^3+^ signal of insulin with its sodium adducts and the [M + 4H]^4+^ signal of the conjugate of insulin and Mal-PEG_12_-DSPE.

In insulin-containing liposomes, lipids containing maleimide and active ester functions can potentially cross-react during API encapsulation. Similar to vancomycin, analysis of insulin encapsulated in Tfp-dPEG_13_-DSPE-containing liposomes revealed substantial derivatization caused by the activated lipid, which was estimated at 12%. Additionally, considerable amounts of lipid-insulin conjugates were also detected for insulin encapsulated in Mal-dPEG_12_-DSPE-containing liposomes, with an estimated modified fraction of 9%. The insulin conjugates with Tfp-dPEG_13_-DSPE (Figure 3A) and Mal-dPEG_12_-DSPE (Figure 3B) were detected with a monoisotopic *m/z* of 7205.275 and 7302.219 (calculated 7205.563 and 7302.601), respectively, confirming the conjugates’ identities. When disulfide functions of macromolecular drugs react with the maleimide group of such activated phospholipids, a free thiol function remains on the API (see Figure 1) that can undergo further side reactions, including the catalysis of disulfide shuffling, which will result in the disintegration of the total encapsulated API.

## 3. Discussion

Already after 45 min of liposomal preparation, the determined quantities of modified API in this study were substantial. Therefore, our findings clearly demonstrate that preparation of liposomes incorporating the highly utilized maleimide and especially active ester activated lipids, either for retrospective coupling or sourcing from insufficient purification of lipid-conjugates, is incompatible with the encapsulation of drugs bearing reactive functional groups such as peptides (and likely proteins and other macromolecular drugs). Because liposomal preparation is predominantly performed at neutral pH due to stability requirements, amino and disulfide functions of encapsulated therapeutics can readily react with active ester and maleimide groups present [31]. 

The extent of unwanted API-modification needs to be considered in regard to the molar amount of API in the formulation and the number and reactivity of the functional groups. For vancomycin containing liposomes, the molar amount of API in our studies was 200-fold larger than that of the insulin containing liposomes. While vancomycin has two amino functions prone to reaction with active esters, insulin has three of them. However, this is not taking into account the different reactivity of the amino functions but should still constitute a sufficiently robust estimate for the comparison of the amounts of reactive functions present in the APIs. Therefore, the amount of functional groups susceptible towards modification in the APIs is approximately 130-fold higher in the vancomycin containing liposomes. The fraction of modified API was substantially higher for insulin containing liposomes (12%) compared to those encapsulating vancomycin (2%). The estimated fraction of modified vancomycin corresponds to a molar amount of 35 µmol. In comparison, the estimated amount of insulin modified was 1.1 µmol, which corresponds to a ratio of modified vancomycin/insulin of approximately 32. This is smaller than the ratio of reactive functions but of a similar magnitude, indicating comparable reaction rates. As a consequence, the unwanted API-modification may be of higher matter for formulations with high lipid-to-drug ratios.

In our liposomal preparations, the lipid-to-drug ratio was 7.2 for vancomycin (molar activated lipid-to-drug ratio 0.036), representing the far lower end of lipid-to-drug ratios for liposomal formulations of macromolecules due to the exceptionally high solubility of vancomycin, while the lipid-to-drug ratio was 360 (molar activated lipid-to-drug ratio 7.1) for the encapsulation of insulin, which is representative for lipid-to-drug ratios of low-soluble macromolecules. Because of the usually high lipid-to-drug ratio of liposomal formulations of macromolecules, the observed considerable amount of API modification is comprehensible. Common lipid-to-drug ratios are >>10 and often reach up to >100, while the portion of activated lipids for retrospective surface modification is usually ≥1%. Therefore, comparable amounts of reactive lipids and encapsulated API can be present in the liposomes (Table 2) causing the considerable extent of side reaction. Additionally, the activated lipids are localized at the liposomal bilayer, resulting in an enhanced local concentration and therefore fostering high reaction rates.

The considerable extent of side reaction of activated lipids with the encapsulated API impacts the reliability of preclinical results of thus prepared delivery systems due to possible variance in the bilayer composition and especially because of the modification of the investigated API. The variance in the bilayer composition may be a consequence of the side reaction of the activated lipids with the encapsulated API, which also reduces the amount of activated lipids available for surface modification. This can therefore result in less amount of surface modification as originally intended. Furthermore, deviations in timing of initiation of the surface modification may cause variances in its amount, due to an ongoing side reaction. The modification of the API decreases its content in the formulation and will proceed until the activated lipids reactivity is quenched (reaction with the surface modification agent). Additionally, the API modification may result in a decrease or complete loss of the API’s potency and can result in adverse effects. In the case of maleimide-activated lipids encapsulating disulfide-connected macromolecules, the observed reaction with the API generates free thiol groups that can catalyze the disintegration of the complete API by disulfide shuffling (especially at neutral to basic pH) and thus should be avoided by all means.

Although possible, lipid modification of therapeutics must not necessarily cause toxicity or loss in potency. In principle, the lipid modification can prolong the half-life of the therapeutic, which may be beneficial for its pharmacodynamics effects (and the monitoring of those in experiments), and can enhance their suitability of encapsulation in liposomes due to incorporation in the bilayer with concurrently decreased aqueous solubility [13]. Nevertheless, a modified API constitutes a new chemical entity and thus a novel API and would therefore require to be characterized as such. If effects cannot definably be traced back to the API under investigation, which is the case for formulations including substantial amounts of modified API, the reliability and transferability of thus obtained preclinical results is hampered. Furthermore, preliminary toxicological evaluation may also be unreliable. This is because the authorities request high purity and defined contents of all components being present in nanocarrier formulations, not only the API. Therefore, for the clinical translation of functional surface-modified delivery systems established on the basis of retrospective coupling utilizing activated lipids, they will have to be re-developed concerning their preparation and will have to be preclinical characterized again.

In this study, the amount of modified API during liposomal preparations comprising activated lipids intended for retrospective coupling of surface modifications was estimated by the performed mass spectrometric analyses. The reason for this approach is that the resulting lipid-peptide conjugates are not readily accessible for quantitative analysis by liquid chromatography-mass spectrometry methodologies due to their ambivalent physicochemical characteristics. Nevertheless, the direct comparison of the absolute intensities of the intact and modified API is anticipated to be sufficiently accurate for estimating the fraction of modified API. Because the API modification is not revealed by standard liposomal characterization using dynamic light scattering (see Table 1) or further characterization techniques such as cryogenic transmission electron microscopy imaging and others, we highly recommend that chemical synthesis of purified lipid-conjugates of the intended surface modification agent is performed prior to liposomal preparation. This already utilized procedure, especially for the preparation of PEGylated liposomes such as Doxil^®^, prevents cross-reactions and assures reproducible and well-defined liposomal formulations. As an alternative, the described post insertion technique can be used. Therefore, one of such “non-reactive” modification strategies should be applied for the plentitude of possible liposomal modifications published.

## 4. Materials and Methods

### 4.1. Materials

Lecithin was obtained from AppliChem GmbH (Darmstadt, Germany), glass beads (0.75–1.0 mm) were purchased from Carl Roth GmbH & Co. KG (Karlsruhe, Germany), NAP™-5 columns were obtained from GE Healthcare (Buckinghamshire, UK) and cholesterol and all solvents were purchased from Sigma-Aldrich (Taufkirchen, Germany). Head group-functionalized phospholipids (2R)-3-((((4,46-dioxo-46-(2,3,5,6-tetrafluorophenoxy)-7,10,13,16,19,22,25,28,31,34,37,40,43-tridecaoxa-3-azahexatetracontyl)oxy)(hydroxy)-phosphoryl)oxy)propane-1,2-diyldistearate(Tfp-dPEG_13_-DSPE) and (2R)-3-((((46-(2,5-dioxo-2,5-dihydro-1H-pyrrol-1-yl)-4,44-dioxo-7,10,13,16,19,22,25,28, 31,34,37,40-dodecaoxa-3,43-diazahexatetracontyl)oxy)-(hydroxy)phosphoryl)oxy)propane-1,2-diyl distearate (Mal-dPEG_12_-DSPE) were purchased from Iris Biotech GmbH (Marktredwitz, Germany) supplied by Quanta Biodesign Ltd. (Plain City, OH, USA). Purified water was produced using an arium^®^ mini (Sartorius, Göttingen, Germany) ultrapure water system. Acetonitrile (ACN), formic acid (FA), and methanol (MeOH), were purchased in the highest available purity from Biosolve (Valkenswaard, the Netherlands).

### 4.2. Methods

#### 4.2.1. Liposomal Preparation

All liposomal formulations were prepared by the film method with subsequent dual asymmetric centrifugation using a SpeedMixer™ (DAC150FVZ Hauschild Engineering GmbH & Co. KG, Hamm, Germany) as previously described [32,33]. Briefly, all lipids required for the respective formulation were dissolved in chloroform/methanol 9:1 (*v/v*) to obtain stock solutions (100 mM). Afterwards, the respective lipids were transferred into Eppendorf tubes in the required amounts (17 mg of lecithin, 1 mg of cholesterol, and 0.4 mg of activated lipid). Subsequently, the organic solvent was evaporated by a nitrogen stream. The resulting lipid film was dried for at least 1 h in a vacuum chamber. Before starting the speedmixing process, about 50 mg of glass beads were added. All liposomal formulations were prepared by speed-mixing at 3500 rpm in a dual asymmetric centrifuge using a special vial holder as described previously [34]. Three runs were performed, and the respective amounts of phosphate buffer and peptide solution were added (Table 3). Precisely, in the first speedmixing round, 50 µL of the respective API were added to the dried lipid film. While vancomycin was dissolved in phosphate buffer (10 mM) at a concentration of 50 mg/mL, insulin was first predissolved in hydrochloric acid (0.1 M) and subsequently adjusted with phosphate buffer (10 mM) to a concentration of 1 mg/mL. In the second and third speedmixing run, only phosphate buffer (10 mM) was added (70 µL and 130 µL).

#### 4.2.2. Liposomal Characterization

The particle size, polydispersity index (PDI) and zeta potential of all liposomal formulations were determined at room temperature using a Zetasizer Nano ZS from Malvern™ (Malvern Instruments Ltd., Worcestershire, UK). Size and PDI were measured after dilution to a lipid concentration of 0.076 mg/mL with a 10 mM phosphate buffer (pH = 7.4) using the automatic mode. The zeta potential was determined after dilution to a lipid concentration of 0.95 mg/mL by a 50 mM phosphate buffer (pH = 7.4). The default settings of the automatic mode of the Zetasizer Nano ZS were: number of measurements = 3; run duration = 10 s; number of runs = 10; equilibration time = 60 s; refractive index solvent 1.330; refractive index polystyrene cuvette 1.590; viscosity = 0.8872 mPa×s; temperature = 25 °C; dielectric constant = 78.5 F/m; backscattering mode (173°); automatic voltage selection; Smoluchowski equation.

#### 4.2.3. Mass Spectrometric Analysis

Liposomal formulations were investigated on a Xevo G2-XS QTof mass spectrometer (Waters, Milford, MA, USA) equipped with a heated Z-Spray electrospray ionization source. Liposomes were disintegrated by treatment with MeOH/Water (3/1; *v/v*) and the solution directly infused into the ion source for analysis combined with a flow of 100 µL of ACN/H_2_O (1/1; *v/v*) + 0.1% FA. Mass spectrometric parameters were as follows: capillary voltage 3000 V, cone voltage 10 V, source temperature 120 °C, desolvation gas flow 600 L/h, and desolvation temperature 250 °C.

## 5. Conclusions

Our findings clearly demonstrate that the risk for potential cross-reactions between activated lipids and macromolecular APIs during liposomal preparation is non-negligible. Substantial amounts of modified API were detected for liposomal preparations comprising activated lipids for post preparation surface modification with estimated modified fractions affecting 2 to 12% of total API. As a consequence, it is crucial for reliable preclinical results that the functional surface modification of liposomes is performed solely by the incorporation of highly purified phospholipid conjugates of the respective modification agents, either prior to the liposomal preparation or by the post insertion technique.

## Figures and Tables

**Table 1:** Comparison of liposomal characteristics with and without activated lipids and APIs (*n* = 3).

Formulation	Control Liposomes	Liposomes Containing Tfp-dPEG_13_-DSPE	Liposomes Containing Mal-dPEG_12_-DSPE	Vancomycin Liposomes Containing Tfp-dPEG_13_-DSPE	Insulin Liposomes Containing Tfp-dPEG_13_-DSPE	Insulin Liposomes Containing Mal-dPEG_12_-DSPE
Size [nm]	124 ± 5	164 ± 5	137 ± 3	150 ± 3	153 ± 2	149 ± 3
PDI	0.20 ± 0.02	0.24 ± 0.02	0.17 ± 0.03	0.22 ± 0.01	0.20 ± 0.01	0.18 ± 0.03
Zetapotential [mV]	−1.1 ± 0.2	−9.4 ± 0.7	−5.3 ± 0.2	−11.4 ± 0.8	−2.4 ± 0.3	−2.3 ± 0.6

**Table 2:** Comparison of lipid-to-drug ratio of the different liposomal formulations.

Formulation	Concentration of Activated Lipid [mM]	Concentration of API [mM]	Molar Activated Lipid-to-Drug Ratio
Insulin liposomes containing Tfp-dPEG_13_-DSPE	0.25	0.035	7.1
Insulin liposomes containing Mal-dPEG_12_-DSPE	0.27	0.035	7.7
Vancomycin liposomes containing Tfp-dPEG_13_-DSPE	0.25	6.9	0.036

**Table 3:** Preparation of liposomes by the speedmixing process.

Speedmixing Run	Duration [min]	Added Volume
1	30	50 µL of the respective API
2	5	70 µL of phosphate buffer
3	1	130 µL of phosphate buffer
